# Prevalence and Predictors of Post-stroke Cognitive Impairment among Stroke Survivors in Uganda

**DOI:** 10.21203/rs.3.rs-2456615/v1

**Published:** 2023-01-16

**Authors:** Martin. N Kaddumukasa, Mark Kaddumukasa, Elly Katabira, Nelson Sewankambo, Lillian. D Namujju, Larry. B Goldstein

**Affiliations:** Makerere University; Makerere University; Makerere University; Makerere University; Makerere University; University of Kentucky

**Keywords:** Stroke, Mild Cognitive impairment, Dementia, Montreal Cognitive Assessment, Prevalence studies

## Abstract

**Background:**

Little is known about the characteristics and determinants of post-stroke cognitive impairments in low- and middle-income countries. The objective of this study was to determine the frequencies, patterns, and risk factors for cognitive impairment in a cross-sectional study of consecutive stroke patients cared for at Uganda’s Mulago Hospital, located in sub-Saharan Africa.

**Methods:**

From August 2019 to July 2020, patients were enrolled a minimum of 3-months post-stroke hospital admission. We collected data on their demographics, vascular risk factors and clinical factors using a questionnaire, clinical examination findings, and test results. Independent predictor variables associated with cognitive impairment were ascertained. Stroke impairments, disability, and handicap were assessed using the National Institute of Health Stroke Scale (NIHSS), Barthel Index (BI), and modified Rankin scale (mRS), respectively. The Montreal Cognitive Assessment (MoCA) was used to assess participants’ cognitive function. Stepwise multiple logistic regression was used to identify variables independently associated with cognitive impairment.

**Results:**

The overall mean MoCA score was 11.7-points (range 0.0–28.0-points) for 128 patients with available data of whom 66.4% were categorized as cognitively impaired (MoCA < 19-points). Increasing age (OR 1.04, 95% CI 1.00-1.07; p = 0.026), low level of education (OR 3.23, 95% CI 1.25–8.33; p = 0.016), functional handicap (mRS 3–5; OR 1.84, 95% CI 1.28–2.63; p < 0.001) and high LDL cholesterol (OR 2.74, 95% CI 1.14–6.56; p = 0.024) were independently associated with cognitive impairment.

**Discussion:**

Further longitudinal, prospective studies are required to confirm these findings and identify strategies for reducing the risk of post-stroke cognitive impairment in this population.

## Introduction

Stroke is a major cause of death and disability among adults worldwide, but particularly in low- and middle-income countries in sub-Saharan Africa (1-3). Stroke is also the second most common cause of cognitive impairment with stroke survivors often experiencing profound cognitive deficits as well as mental health impairments (3). Up to 64% of stroke survivors develop some degree of cognitive impairment and about 30% die with complications from dementia (4, 5). Post-stroke cognitive impairment characteristically involves multiple domains including attention and concentration, executive function, language, memory and visuospatial function, with executive function being the greatest affected (4, 6,7). Persons with mild cognitive impairment (MCI) convert to Alzheimer’s disease (AD) at an annual rate of 10–12% in contrast to 1–2% in the elderly population without MCI (8). Cognitive impairment can have an important impact on quality of life and activities of daily living by reducing independence (6) and is associated with long-term morbidity and disability (9).

Although the prevalence of post-stroke cognitive impairment has been studied in different countries (10, 11), data are inconsistent due to differences in patient characteristics, neuro-psychological assessments, sample sizes, and analytical methods (12). Accurate estimation of the prevalence of post-stroke cognitive impairment is limited by these and other factors with frequencies varying from 30–50% (13). A myriad of factors have been proposed as predisposing to post-stroke vascular cognitive impairment including socio-demographic variables such as age, sex, educational attainment, occupation and environmental enrichment, cardiovascular risk factors, and stroke-related characteristics such as the extent and sites of brain injury (3, 4, 14, 15). There are only limited data characterizing the burden, spectrum, determinants and consequences of post-stroke cognitive impairment in patients residing in low- and middle-income countries in sub-Saharan Africa (3). In the present study, we assessed the frequency and risk factors associated with cognitive impairment 3-months after stroke in patients who received care in the primary referral hospital in Uganda.

## Methods

### Study design, setting and population.

This was a cross-sectional study conducted at Mulago Hospital, Uganda’s main national referral hospital. Consecutive patients who had a first-ever or recurrent stroke and were admitted to the hospital between August 2019 - July 2020 were recruited. Stroke was defined as a neurologic deficit of abrupt onset attributable to a vascular cause with neurologic deficits lasting more than 24-hours and with compatible findings on CT brain scan (16, 17). Cognitive impairment was defined as a decline in function in either one or several domains of cognitive function as assessed by the Montreal Cognitive Assessment (MoCA) (18) or indicated on patient medical record.

Study subjects were age > 18-years and had a minor-to-severe stroke (National Institutes of Health Stroke Scale [NIHSS] score between 1–25) and enrolled a minimum of 3-months post stroke admission. Those whose stroke status could not be confirmed on CT scan and those with a history of severe cognitive impairment (dementia) prior to the index stroke, or a history of psychiatric disease (schizophrenia, manic-depressive disorder, and major depression) were excluded (13, 19).

### Data collection.

A questionnaire was administered to collect demographic information including age, highest level of education, occupation, sex, work status, marital status and living conditions. Vascular risk factors were assessed based on self-report, reported use of key medications, and review of medical records. Cardiac disease including myocardial infarction and atrial fibrillation were assessed based on self-reported history, clinical examination, and review of both baseline and historical ECG results.

### Clinical assessments.

All participants had a general physical examination after enrollment. Blood pressure was measured with an automated sphygmomanometer twice after an interval of 15-minutes with the subject seated and the average recorded. Hypertension was defined as BP > 140/90mmHg and/or receiving antihypertensive medications over a period longer than 1-month. Diabetes mellitus was diagnosed based on having a fasting blood glucose level > 126mg/dl and/or receiving related medications. Dyslipidemia was defined as total cholesterol concentration > = 4.5mmol/L, and/or LDL cholesterol > = 2.5mmol/L (20) and/or previous use of statin for dyslipidemia.

Stroke impairment and post-stroke handicap were assessed at enrollment using the NIHSS (21) and modified Rankin scale (mRS), respectively (22, 23) by trained research assistants based on retrospective medical record review and interview of stroke survivors and/or their proxy. NIHSS scores ranging from 1–4, 5–15 and 16–25 were defined as mild, moderate, and severe stroke respectively; mRS scores 0–2 and 3–5 corresponded to mild and severe post-stroke handicaps, respectively.

Assessment of patients’ dependency was based on the Barthel Index (BI) obtained on the day of enrollment and classified into three grades: Complete dependence (BI score < 60), moderate dependence or assisted independence (BI score 60–94), and minimal or no disability (BI score ≥ 95)(24).

The Montreal Cognitive Assessment (MoCA) (25, 26) was used to assess participants’ cognitive function. The MoCA includes eight cognitive domains (attention, concentration, memory, language, orientation, visuo-constructional skills, conceptual thinking, and calculation). Responses were scored according to specified criteria, with a maximum score of 30 (13, 27). Scores < 19 and > = 19 were categorized as reflecting cognitive impairment and no impairment, respectively. An optimal cutoff of 19 for the detection of mild cognitive impairment among minority Blacks with low level of education of < 12 years has been proposed (28). To ensure the validity and reliability of the MoCA test, it was administered after rigorous training sessions with Luganda (local language)-speaking examiners, conducted with discussions with an expert panel of 2 neurologists, 1 psychologist and 1 educator. The phonemic fluency task and sentence repetition task were deemed problematic; thus were substituted a semantic fluency task (animal naming) for phonemic fluency, a strategy also used in the Korean version of the MoCA (18).

### Statistical analysis.

Descriptive statistics including means, frequencies and percentages were used to summarize participants’ cognitive data over different socio-demographic variable groups including age, sex, education level, living conditions, and work status. Univariable associations between the outcome variable, cognitive impairment, and categorical baseline variables were determined with chi-square tests; t-tests were used for continuous variables. Participant records with key missing data were dropped from the analysis. The results of the univariable analyses were subsequently adjusted for known confounders including age, sex and stroke severity (13) in a stepwise multiple logistic regression model using both forward and backward elimination to determine independent relationships with cognitive impairment.

## Results

**Figure 1:**
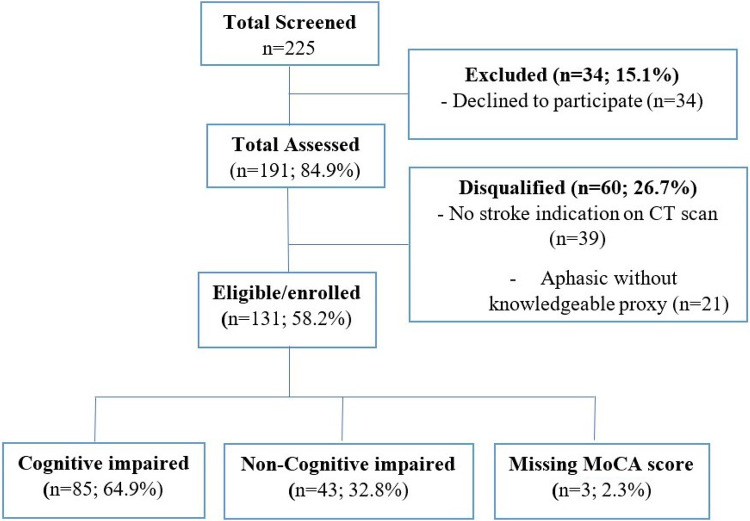
Study Consort Flow Diagram

131 stroke patients were enrolled (age range 20 to 91-years) including 72 (54.2%) women. Figure 1 shows the consort flow diagram.

Participants were enrolled a mean of 3-months post-index stroke. The majority (71.8%) had an ischemic stroke and 28.1 % had a hemorrhagic stroke. A total of 46.6% were retired or not working and 47.3% reported either living alone or with distant relatives. The average age of participants was 57.8 ± 14.7 years with 56.5% younger than 60-years; 50.4% reported a minimum of a high school education and higher. Table 1 gives the demographic and clinical characteristics of the study population.

### Frequency, patient profiles and patterns of cognitive performance.

**Figure 2:**
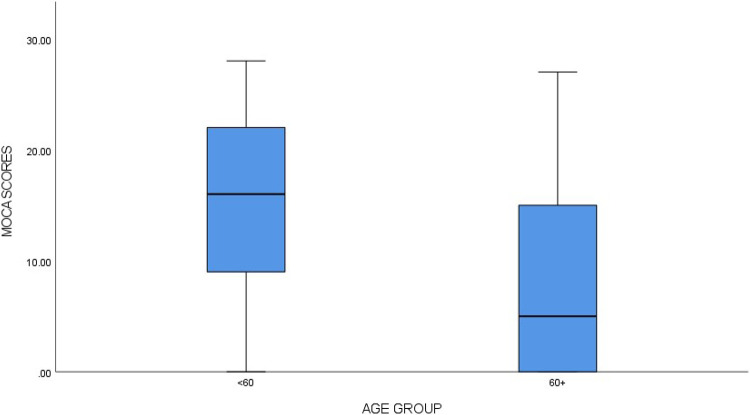
MOCA scores by Age group

The MoCA was completed by 128 (97.7%) participants. MoCA scores ranged between 0.0–28.0 points with an average score of 11.7-points and mode of 0.0-points. Cognitive impairment was detected in 85 (66.4%), with 43 (33.6%) classified as having no cognitive impairment. Figure 2 shows MoCA score distribution for age groups < 60 and 60 + years.

70.0% of the female study participants were cognitively impaired compared to 62.1% of male participants. In addition, 69.4% of the illiterate or poorly educated population were women compared to 30.6% of men (OR (3.27, 95% CI 1.57,6.8; p = 0.001).

Several variables were associated with cognitive impairment in univariate analyses including age >60*yrs* (p < 0.0001), high LDL-C (p = 0.048), lower education (p < 0.0001), not working or retired status (p = 0.002), living alone or with relatives compared with spouse/partner (p = 0.005), higher mRs score (p = 0.001), Barthel scores corresponding to severe to total dependence scores (p < 0.0001), and recurrent stroke (p = 0.014). Table 2 gives the results of the univariate analyses with cognitive impairment as the dependent variable.

After adjustment for age, sex and stroke severity in a multivariate analysis, low education (p = 0.016), high mRS scores (p < 0.001) and high LDL-C (p = 0.024) remained significantly associated with moderate to severe cognitive impairment (Table 3).

## Discussion

We found a high frequency of post-stroke cognitive impairment assessed a minimum of 3-months after stroke in a consecutive series of patients evaluated at the primary referral hospital in Uganda. The overall frequency of 66.4% is higher than a previous 43% estimate in this population (29). The previous study, conducted in 2005, involved a smaller sample of participants (n = 77). Our result is consistent with findings in Nigeria (67.4%), Ghana (72.8%), Korea (62.6%), and Norway (52.1%) (3, 30, 31). A meta-analysis of 65 observational studies, published in 2019, reported sparse data on cognitive impairment in sub-Saharan Africa, but found overall prevalence among African people aged 5- years or older at 30–40% (32). The high prevalence may be because most stroke patients in this population present late to health facilities and to the lack of cognitive rehabilitation intervention availability in Uganda’s existing post stroke management care protocols.

In multivariable analyses, we adjusted for variables associated with cognitive outcomes in univariable analyses and for potential confounders identified in other studies (i.e., age, sex, and stroke severity) (22). Our univariate analysis found that age > 60-years was associated with post stroke cognitive impairment, consistent with prior studies in other populations (20). In contrast to some other studies (33, 34), we found no associations between sex and stroke severity (as measured by NIHSS scores) with MCI. The lack of associations may be related to the timing of our assessments, the type of cognitive assessments, and differences in educational attainment between study populations. The extent of formal education is protective against cognitive impairment in vascular dementia, Alzheimer’s disease and mild cognitive impairment (3, 35). A higher educational level increases the brain’s cognition reserve, which may lead to improved compensation with aging and brain injury (20).

We found higher LDL-C was associated with greater cognitive impairment. This result is consistent with a previous cross-sectional analysis from four U.S. cities which found a positive association between LDL-C levels and cognitive measures (10, 36). Additionally, findings from the Northern Manhattan Stroke Study showed that higher LDL-C was associated with the risk of incident vascular dementia (10, 37). The mechanisms underlying the association between LDL-C and cognitive impairment are unknown, and different explanations have been proposed (8, 38, 39). A recent study showed that LDL -C activates the secretion of pro- inflammatory mediators such as tumor necrosis factor alpha and interleukin-6 and decreased the BBB membrane injury, a contributory factor to the development and progression of cognitive impairment (40, 41).

High mRS score (i.e., 3 - 5) was independently associated with cognitive impairment. Scores 3–5 are associated with high to total patient handicap and reliance on others in performing activities of daily living (42). The impact of a stroke goes far beyond physical disability (43), impairing higher-order cognitive functions such as motor control, organization, problem solving, and memory. Stroke survivors who are physically dependent and more impaired tend to perform poorly in these tasks (43).

Patient functional dependence as assessed by the BI predicted cognitive impairment as a single variable; however, the association between BI and cognitive impairment attenuated as was not significant when controlling for potential confounding variables in the multivariate analysis. This could be due to collinearity between the independent variables or it could also be a result of the BI instrument. It has been postulated that functional dependence is not a pure personal characteristic but is instead a gap between one’s ability and one’s personal needs suggesting the existence of other environmental, social, and individual adaptations in contributing to activities of daily living (44). In addition, the sensitivity of the BI to distinguish between high and low performers is limited (45). It is not efficient in detecting problems in more complex social skills as it only involves 8 basic daily life tasks. Only severe cognitive impairment may interfere with these tasks (45).

Our study has several limitations. Our relatively small sample size could reduce our statistical power to identify associations of lower effect size. Because of the cross-sectional design, we cannot provide evidence of causal relationships between putative predictors and post-stroke cognitive impairment. Dementia involves a change in function and cognition, but we did not conduct longitudinal assessments and in some cases, relied on proxy reports for some variables (3). There is also a potential inception cohort bias because our sample included only participants seen at a single national referral hospital. The MoCA is not validated in a sub-Saharan African population and therefore does not take account of certain linguistic and cultural variables which may affect interpretation of the MoCA across different countries. In addition, participant level of education affects the results of some screening tests, including the MoCA, resulting in a possible overestimation of cognitive impairment among those who are illiterate. Using a MoCA cut-off of 19 to classify cognitive impairment in this population rather than the more common 26 points, however, mitigates this effect. (4) Most of the study participants had a relatively mild stroke and our results may not be generalizable to those with a more severe or very mild stroke, or to those who have specific deficits (e.g., aphasia) that precluded cognitive assessment.

Limitations notwithstanding, this study provides unique data on the frequency and risk factors for poststroke cognitive impairments in Uganda, a resource constrained country in sub-Saharan Africa. The data are helpful for creating public awareness, influencing policy recommendations, and guiding further research that may lead to effective interventions.

## Figures and Tables

**Table 1:** Study Group Characteristics

Characteristic	Frequency	Percentage (%)
Overall	131	100%
Mean, Mode, Median, [Range]		
*MoCA Score*	11.7, 0.0, 13.0; [0.0–28.0]	
*NIHSS Score*	11.0, 10.0, 10.0; [0.0–30.0]	
*MRS score*	3.3, 4.0, 4.0; [0.0–5.0]	
Mean (SD), [Range]		
Age (Years)	57.8(14.7); [20–91]	
*HDL*	1.5(0.8); [0.5–4.8]	
*LDL*	2.9(1.1); [0.8–5.7]	
*CHOL*	4.0(1.3): [1.6–7.4]	
*TG*	2.1(0.9); [1.0–4.8]	
**Age group (years)**		
*< 45*	20	15.3%
*45–60*	54	41.2%
*> 60*	57	43.5%
**Cognitive Impairment**		
*No*	43	33.6%
*Yes*	85	66.4%
**Gender**		
*Female*	72	54.90%
*Male*	59	45.04%
**Education**		
*High*	66	50.40%
*Low*	65	49.60%
**Marital Status**		
*Married*	79	60.30%
*Single/Divorced/Widowed*	52	39.70%
**Work Status**		
*Not Working*	61	46.60%
*Working*	70	53.40%
**Living Status**		
*Alone/with relatives*	62	47.30%
*With Partner/Spouse*	69	52.70%

**Table 2:** Results of Univariate Analysis, Cognitive Impairment Crude RR by characteristics

Prevalence of Cognitive Impairment, Crude RR by characteristics			
Clinical Characteristics	No. of Cognitive impaired (%)	Crude OR, 95% CI	p- value
**Overall**	85/128 (66.4%)		
Sex: *Female*	49/70(70.0%)	1	
Sex: *Male*	36/58(62.1%)	0.70(0.34,1.47)	0.344
Age: *<60 years*	39/73(53.4%)	1	
Age: *60 + years*	46/55(83.6%)	4.46(1.91,10.42)	< 0.0001
*High Education*	34/66(51.5%)	1.00	
*Low Education*	51/62(82.3%)	4.36(1.94,9.82)	< 0.0001
*Status: Married*	47/78(60.3%)	1.00	
*Status: Single/Divorced/Widowed*	38/50(76.0%)	2.09(0.95,4.61)	0.066
*Work status: Working*	37/68(54.4%)	1.00	
*Work status: Not Working*	48/60(80.0%)	3.36(1.52,7.41)	0.002
*Living status: Alone/with relatives*	48/61(78.7%)	1.00	
*Living status: With Partner/Spouse*	37/67(55.2%)	0.33(0.15, 0.73)	0.005
*NIH score: Minor to no stroke (0–4)*	31/45(68.9%)	1.00	
*NIH score: Moderate to severe stroke (5–20)*	54/83(65.1%)	0.84(0.39,1.83)	0.661
*MRS score: Good Outcome (0–2)*	14/34(41.2%)	1.00	
*MRS score: Poor Outcome (3–5)*	62/83(74.7%)	4.22(1.82,9.80)	0.001
*Barthel score: Mild to no dependence (12–20)*	29/59(49.2%)	1.00	
*Barthel score: Severe to total dependence (< 12)*	56/69(81.2%)	4.46(2.02,9.80)	< 0.0001
*Stroke type: Hemorrhagic*	22/37(59.5%)	1.00	
*Stroke type: Ischemic*	63/91(69.2%)	1.53(0.69,3.39)	0.289
*High BP: No*	50/70(71.4%)	1.00	
*High BP: Yes*	35/58(60.3%)	0.61(0.29,1.27)	0.186
*Recurrent stroke: No*	61/100(61.0%)	1.00	
*Recurrent stroke: Yes*	24/28(85.7%)	3.84(1.24,11.90)	0.014
*Atrial Fibrillation: No*	70/110(63.6%)	1.00	
*Atrial Fibrillation: Yes*	15/18(83.3%)	2.86(0.78,10.47)	0.101
*Cholesterol: Low*	63/97(64.9%)	1.00	
*Cholesterol: High*	22/31(71.0%)	1.32(0.55,3.18)	0.537
*LDL: Low*	30/53(56.6%)	1.00	
*LDL: High*	55/75(73.3%)	2.11 (1.00,4.44)	0.048

**Table 3:** Results of Multi-variate Analysis, Cognitive Impairment, Adjusted Crude RR by characteristics

Prevalence of Cognitive Impairment, Adjusted Crude RR by characteristics			
Clinical Characteristics	No. of Cognitive impaired (%)	Adjusted* Crude OR, 95% CI	*p- value
**Overall**	85/131 (64.9%)		
**Education**			
*High*	34/66(51.5%)	1	
*Low*	51/62(82.3%)	3.23(1.25,8.33)	0.016
**Marital Status**			
*Married*	47/78(60.3%)	1	
*Single/Divorced/Widowed*	38/50(76.0%)	1.69(0.65,4.35)	0.285
**Work Status**			
*Working*	37/68(54.4%)	1	
*Not Working*	48/60(80.0%)	1.86(0.68,5.08)	0.228
**Living Status**			
*Alone/with relatives*	48/61(78.7%)	1	
*With Partner/Spouse*	37/67(55.2%)	0.46(0.19,1.15)	0.096
**MRS score**			
*Good Outcome (0–2)*	14/34(41.2%)	1	
*Poor Outcome (3–5)*	62/83(74.7%)	1.84(1.28,2.63)	< 0.001
**Barthel Score**			
*Mild to no dependence (12–20)*	29/59(49.2%)	1	
*Severe to total dependence (< 12)*	56/69(81.2%)	2.68(0.94,7.70)	0.067
**Stroke Type**			
*Hemorrhagic*	22/37(59.5%)	1	
*Ischemic*	63/91(69.2%)	1.03(0.41,2.56)	0.953
**High BP**			
*No*	50/70(71.4%)	1	
*Yes*	35/58(60.3%)	0.69(0.30,1.61)	0.397
**Recurrent stroke**			
*No*	61/100(61.0%)	1	
*Yes*	24/28(85.7%)	3.57(0.91,14.29)	0.068
**Atrial Fibrillation**			
*No*	70/110(63.6%)	1	
*Yes*	15/18(83.3%)	1.09(0.24,4.98)	0.911
**Cholesterol**			
*Low*	63/97(64.9%)	1	
*High*	22/31 (71.0%)	2.47(0.86,7.07)	0.093
**LDL**			
*Low*	30/53(56.6%)	1	
*High*	55/75(73.3%)	2.74(1.14,6.56)	0.024

* Adjusted for age, sex and stroke severity
